# Antibody-targeted T cells and natural killer cells for cancer immunotherapy

**DOI:** 10.1186/s12951-024-02898-3

**Published:** 2024-10-18

**Authors:** Ashley R. Sutherland, Brijesh Parlekar, David W. Livingstone, Andrés X. Medina, Wendy Bernhard, Tays Hernández García, John DeCoteau, C. Ronald Geyer

**Affiliations:** 1 Department of Biochemistry, Microbiology and Immunology, University of Saskatchewan, Saskatoon, SK S7N 5E5 Canada; 2 Department of Health Sciences, University of Saskatchewan, Saskatoon, SK S7N 5E5 Canada; 3 Department of Pathology and Laboratory Medicine, University of Saskatchewan, Saskatoon, SK S7N 5E5 Canada; 4 Center for Molecular Immunology, 11600 Havana, Cuba

## Abstract

**Background:**

Adoptive cell cancer therapies aim to re-engineer a patient’s immune cells to mount an anti-cancer response. Chimeric antigen receptor T and natural killer cells have been engineered and proved successful in treating some cancers; however, the genetic methods for engineering are laborious, expensive, and inefficient and can cause severe toxicities when they over-proliferate.

**Results:**

We examined whether the cell-killing capacity of activated T and NK cells could be targeted to cancer cells by anchoring antibodies to their cell surface. Using metabolic glycoengineering to introduce azide moieties to the cellular surface, we covalently attached a dibenzocyclooctyne-modified antibody using the strain-promoted alkyne azide cycloaddition reaction, creating antibody-conjugated T and NK cells. We targeted the immune cells to tumors possessing the xenoantigen, N-glycolyl neuraminic acid GM3 ganglioside, using the 14F7hT antibody. These activated T and NK cells are “armed” with tumour-homing capabilities that specifically lyses antigen-positive cancer cells without off-target toxicities. Moreover, when exposed to target cells, 14F7hT-conjugated T cells that are not preactivated exhibit increased perforin, granzyme, CD69, and CD25 expression and specific cell killing.

**Conclusions:**

This research shows the potential for a non-genetic method for redirecting cytotoxic immune cells as a feasible and effective approach for tumor-targeted cell immunotherapy.

**Graphical Abstract:**

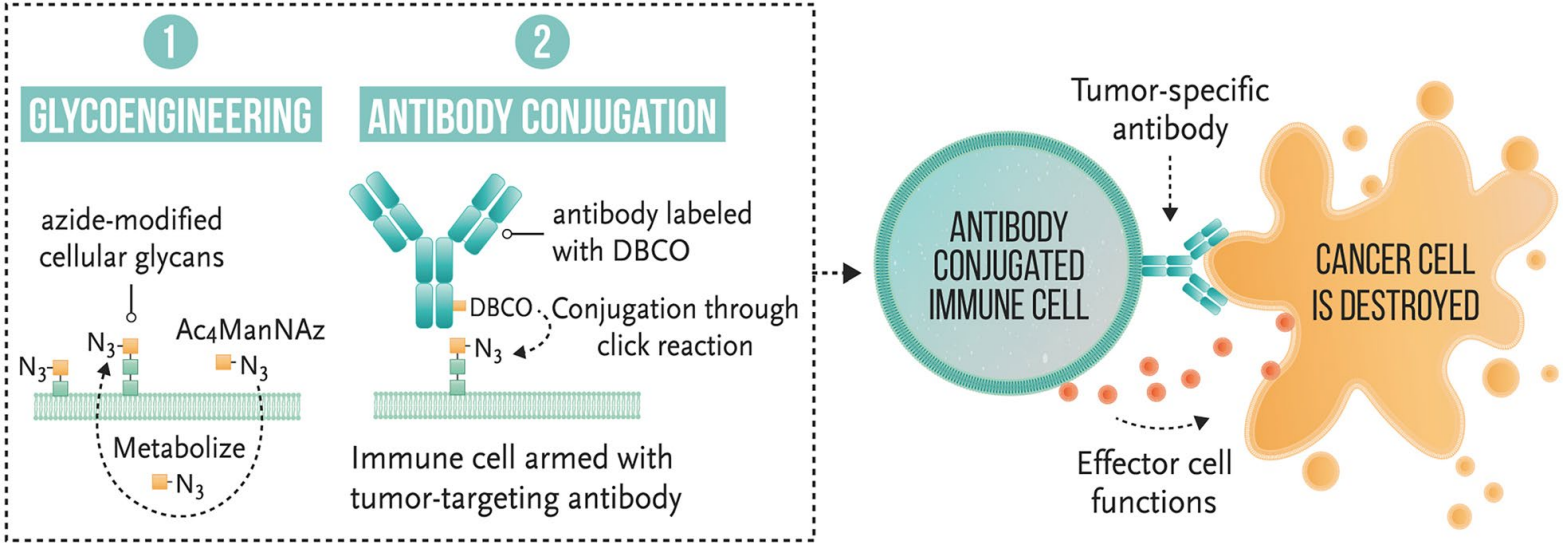

**Supplementary Information:**

The online version contains supplementary material available at 10.1186/s12951-024-02898-3.

## Introduction

Cell membrane engineering has a wide range of applications in both basic and applied science, from the study of cell signaling and cell–cell interactions to the recent advancement of cell therapies. Chimeric antigen receptor (CAR) T and NK cell therapies rely on the expression and engineering of cancer-antigen binding chimeric receptors on the surface of immune cells through genetic manipulation [1, 2]. CAR cell therapies have shown remarkable success in treating a variety of hematopoietic cancers; however, rapid expansion of engineered cells can induce severe cytokine release and neurotoxic side effects [3–5]. Furthermore, the incorporation of CAR in immune cells through viral vectors has constraints such as technical difficulties and safety concerns [6, 7]. These include heterogenous CAR expression levels, inconsistent viral transduction efficiency, and T cell tumorigenicity caused by the random integration of the viral genome into the host chromosome, all at a significant financial and technical cost to produce [7, 8]. Consequently, new chemistries have been developed for the direct attachment of molecules to the surface of cells [9, 10]. Nanomaterials, ligands, small molecules, DNA, and artificial receptors have been anchored to the cellular surface to augment the functionality of the cell for applications such as drug delivery, immunotherapy, cancer imaging, tissue homing, and immune evasion [9–12].

Strain-promoted azide-alkyne cycloaddition (SPAAC), a form of copper-free click chemistry, has been widely employed for cell engineering and bioconjugation and has become an attractive technique in the field of biomedicine due to its excellent specificity, high reaction rate, small molecule size, and biocompatibility [13]. Metabolic azide glycoengineering modifies cellular glycans with unnatural azide moieties, which can be exploited for subsequent conjugation with cyclooctyne-containing functional groups [13]. Pairing metabolic glycoengineering with SPAAC enables the conjugation of alkyne modified molecules to the cell surface, conferring new attributes to cells without the need for genetic manipulation.

We sought to investigate strategies to engineer activated T cells and NK cells with a targeting antibody to direct them toward tumors with high specificity and reduced potential off-tumor effects. 14F7hT [14] is a well-characterized antibody with promising clinical potential, specifically recognizing N-glycolyl neuraminic acid (Neu5Gc) GM3 ganglioside, an antigen found in the cellular membrane of a variety of cancers; however, it is absent from healthy human tissues, making it an attractive target for immunotherapy [15, 16]. Neu5Gc is not produced in normal human cells due to a deletion in the gene encoding the enzyme converting N-acetylneuramic acid (Neu5Ac), a common component of cellular membranes, to Neu5Gc [17]. In contrast to most mammals, Neu5Gc is considered a xenoantigen in humans, with malignant cells metabolically incorporating the antigen from dietary sources as a result of increased metabolic rate and hypoxia-induced upregulation of sialic acid transporter, sialin [18].

Cell membrane engineering of NK and T cells has allowed the redirection of cytotoxic immune cells towards tumors. Activated T cells have been targeted via glycoengineering to attach targeting ligands [19] as well as aptamers [20] for tumor-specific killing. NK cells have been equipped with ligands through glycoengineering [21, 22], with lipid-anchored [23] and glycoengineered cells with aptamers [24] as well as antibodies [25] and nanobodies [26] attached enzymatically. Expanding on the work by Wang et al*.* who used 9-azido *N*-acetyl neuraminic acid methyl ester to metabolically glycoengineer NK-92 cells for subsequent conjugation to DBCO-modified Cetuximab [27], we expanded the use of glycoengineering and SPAAC chemistry to label both T and NK cells with tumor-targeting antibodies and compared the tumor-lysing potential of both cell types. We expanded our study to include unactivated T cells, demonstrating the utility of glycoengineering and bioorthogonal reaction to redirect cytotoxic cells with potential clinical applications.

## Materials and methods

### Labelling of antibody with DBCO-PEG_4_-NHS ester

14F7hT antibody was obtained from Center of Molecular Immunology (Havana, Cuba). IgG antibody was labelled non-specifically with DBCO-PEG_4_-NHS ester (Click Chemistry Tools) using a molar ratio of 3:1 labelling reagent to antibody. The reaction mixture was incubated at room temperature with shaking (600 rpm) for 1 h. Excess labelling reagent was removed by Zeba Spin 40 kDa molecular weight cut-off desalting column (Thermo Fisher Scientific) and centrifuged at 1000xg for 4 min. Protein concentration and degree of labelling were determined by measuring A_280_ and A_309_ using a NanoDrop 2000c spectrophotometer (Thermo Fisher Scientific).

### Cell culturing

Primary human T cells were obtained from healthy donors using an Institutional Review Board-approved protocol. Human peripheral blood mononuclear cells (PBMCs) were isolated from whole blood using Ficoll-Hypaque (Sigma). CD4^+^/CD8^+^ T cells were isolated with positive magnetic selection (StemCell Technologies) with a 1:1 ratio of CD4^+^/CD8^+^ and cryopreserved for future use with 90% fetal bovine serum (FBS) (Sigma) and 10% dimethyl sulfoxide (DMSO) (Sigma). T cells were cultured in ImmunoCult (StemCell Technologies) supplemented with 100 IU/mL human recombinant IL-2 (StemCell Technologies) and 100 IU/mL penicillin/streptomycin (Gibco). Activated T cells were expanded for 6 days with ImmunoCult anti-CD3/CD28 antibody activation beads (StemCell Technologies) added on Day 1 and Day 5. Non-activated cells were not expanded prior to use in experiments.

Cell lines were purchased from American Type Culture Collection (ATCC) and maintained in media and 100 IU/mL penicillin/streptomycin. L1210 was maintained in Dulbecco's Modified Eagle Medium (DMEM) with 10% horse serum (Gibco), K562 in Roswell Park Memorial Institute (RPMI) 1640 Medium with 10% FBS (Gibco) and NK-92 in Alpha Minimum Essential Medium with 12.5% FBS, 12.5% horse serum, 100 IU/mL IL-2, 0.2 mM Myo-inositol (Sigma), 0.1 mM 2-mercaptoethanol (Gibco) and 0.02 mM folic acid (Sigma).

Incorporation of free Neu5Gc into glycoproteins of K562 cells was achieved through culturing cells in media containing 3 mM Neu5Gc (Cayman Chemical) for 72 h.

### Production and characterization of antibody conjugated T and NK-92 cells

To metabolically glycoengineer the surface of T and NK-92 cells, cells were seeded at a density of 1 × 10^6^ cells per mL in culture medium supplemented with Ac_4_ManNAz (Click Chemistry Tools) for 24 h. To identify the azide group incorporated on the T cell surface, DBCO-conjugated AZDye 488 (Click Chemistry Tools) was incubated with glycoengineered T cells for 30 min at room temperature and quantified by flow cytometry (CytoFLEX Flow Cytometer, Beckman).

To generate AbC T and AbC NK cells, DBCO-labeled antibody was added to cells at 4 μM in PBS + 2% FBS for 2 h at 37 °C at a cell concentration of 10 × 10^6^ cells/mL. Cells were washed twice with PBS then used in experiments.

Conjugation of DBCO-antibody to the surface of glycoengineered T and NK-92 cells was confirmed through staining with PE mouse anti-human IgG (BD Biosciences) and analyzed by flow cytometry. To evaluate biostability, AbC T and AbC NK cells were maintained in culture media and antibody signal (MFI) quantified by flow cytometry at various time points.

Viability of T and NK cells was measured by Trypan blue exclusion assay and cell counts performed using a hemacytometer.

### Cell interaction assay

Target cells were pre-stained stained with CellTrace Violet (CTV, Thermo Fisher Scientific) and activated T or NK-92 cells were labeled with CellTracker Green (CTG, Thermo Fisher Scientific). Effector and target cells (1 × 10^5^ cells of each) were mixed 1:1 ratio in a 96 well round-bottom plate in a total volume of 100 μL Immunocult media. Cells were incubated for 2 h at 37 °C, pipetted gently and analyzed by flow cytometry. Populations of effector cells (CTG+), target cells (CTV+) and E/T clusters (CTV+CTG+) were gated. Percentage of interacting effector cells was calculated by the formula:

$$\frac{\text{E}/\text{T clusters}}{(\text{E}/\text{T clusters}+\text{Effector cells})} \times 100$$

### Cytotoxicity assay

T or NK-92 cells were pre-stained with CellTrace Violet and mixed with 5 × 10^4^ target cells, with varying numbers of effector cells, maintaining effector cell concentration at 1 × 10^6^ cells/mL. Cells were co-cultured for 16 h at 37 °C and stained with 7-AAD cell viability dye (Thermo Fisher Scientific) followed by flow cytometry analysis. Target cell death was determined by analyzing 7-AAD staining of CellTrace Violet negative target cells.

### Chronic lymphocytic leukemia (CLL) patients cell cytotoxicity

Patients were clinically diagnosed based on World Health Organization criteria for CLL [28]. PBMCs from CLL patient blood were obtained using an Institutional Review-approved protocol and were isolated and cryopreserved as previously described. Patients cells were cultured in RPMI supplemented with 10% FBS and 100 IU/mL IL-2.

Engineered activated T or NK cells were mixed with 1 × 10^5^ patient cells at an 8:1 E/T ratio as previously described and incubated for 16 h. To detect the 14F7hT positive population in patient cells, cells were stained with biotinylated 14F7hT for 30 min at room temperature followed by staining with Streptavidin FITC conjugate (Thermo Fisher Scientific) for 30 min on ice. Cells were stained with 7-AAD and target cell death was measured by analyzing 7-AAD staining of 14F7hT positive target cells.

### Activation assay

Unactivated T cells or AbC T cells and target cells were co-cultured with L1210 target cells as previously described at a 4:1 E/T ratio and stained with APC anti-human CD69 (Biolegend), Pacific Blue anti-human CD25 (Biolegend) and FITC anti-human CD3 (Miltenyi Biotec) for 20 min on ice followed by flow cytometry analysis.

### Granzyme/perforin assay

Unactivated T cells or AbC T cells and target cells were co-cultured for 12 h as previously described at a 4:1 E/T ratio in culture media containing GolgiStop protein transport inhibitor (BD Biosciences). Cells were fixed and permeabilized to facilitate intracellular staining of granzyme and perforin using Cytofix/Cytoperm Fixation/Permeabilization kit (BD Biosciences) followed by staining with FITC anti-human perforin (Thermo Fisher Scientific) and APC anti-human granzyme B (Biolegend).

### Co-immunoprecipitation analysis

Ten million T cells or AbC T cells were lysed for 30 min on ice in 200 μL CHAPS lysis buffer supplemented with PIC and PMSF (Sigma). Lysates were centrifuged at 4 °C at 13,000×*g* for 10 min, with 10 μL stored at 4 °C to be used as input material. The remaining 190 μL supernatant was incubated with 50 μL of pre-washed Protein A magnetic beads (Thermo Fisher Scientific) for 1 h with rotation at 4 °C. The beads were washed five times with 1000 μL CHAPS lysis buffer followed by addition of 40 μL elution buffer (20 μL 65 mM glycine, 20 μL 2X sample loading buffer [BioRad] containing 46 mg/mL dithiothreitol) at 70 °C for 10 min. The input and eluted material was evaluated on a 12% Bis–Tris gel and Western blot probing for anti-human CD3ε (Cell Signaling Technology) at a 1:1000 dilution and HRP conjugated anti-rabbit IgG at 1:5000 dilution. The blot was imaged by ChemiDoc MP Imaging System (BioRad).

### Determination of density of antibody on cell surface

NK-92 and activated T cells were conjugated with antibody followed by staining with PE mouse anti-human IgG. The number of antibody molecules per cell was determined using Quantum Simply Cellular anti-Human IgG kit (Bangs Laboratories).

### Statistical analysis

Data is displayed as the mean ± standard deviation with a sample size (n) for statistical analysis of at least three. Statistical analyses were performed using a two-tailed paired *t*-test. *p* values of *<0.05, **<0.01, ***<0.001 and ****<0.0001 were considered statistically significant, ns, no statistically significant difference.

## Results

### Generation of antibody-conjugated T and NK cells

**Fig. 1:**
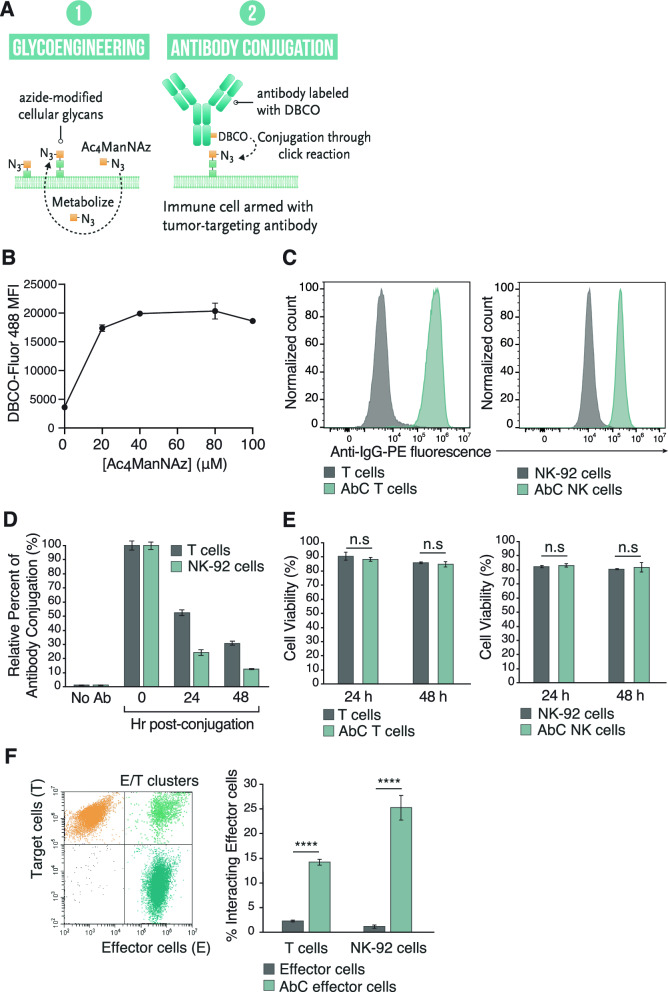
Generation and characterization of AbC T and AbC NK cells. **A** AbC immune cell engineering scheme. **B** Optimization of glycoengineering of activated T cells. T cells were cultured with varying concentrations of Ac_4_ManNAz and azides were detected using DBCO-Fluor 488 with fluorescence quantified via flow cytometry. **C** DBCO-Ab conjugation to glycoengineered T (*left*) and NK-92 cells (*right*) as measured by flow cytometry. **D** Stability time course study of AbC T and AbC NK. Relative percent of antibody on surface was calculated with respect to time 0. **E** Cell viability assay of antibody-conjugated and unconjugated T (*left*) and NK cells (*right*) at 24 and 48 h by Trypan Blue exclusion. **F** Specific binding of AbC T and AbC NK cells to target cells. Effector cells (AbC or unconjugated T/NK) and target cells (L1210) were mixed at 1:1 E/T ratio for 2 h and resulting E/T cluster formation detected by flow cytometry. Cell populations were gated for effector cells, E, (*green fluorescence*), target cells, T, (*violet fluorescence*) and E/T with fluorescent emission of both signals using gating strategy shown on left. Percentage of interacting effector cells was calculated (*right panel*). Data represents the mean ± SD (*n* = 3). (Student’s *t* test, ****p < 0.0001, *ns* not significant)

To endow activated T and NK cells with MHC-independent tumor-targeting capabilities, we covalently attached the 14F7hT tumor targeting antibody to the cellular surface using SPAAC click chemistry. We utilized the clinically relevant NK cell line, NK-92, which shows characteristics of highly active natural killer cells and can be engineered and given to patients with cancer [29, 30]. T cells were activated via culturing with anti-CD3/CD28 antibody activation beads and expanded for 6 days. Activated T cell and NK-92 cell were modified with cell surface azides by culturing them with N-azidoacetylmannosamine-tetraacylated (Ac_4_ManNAz)[31] followed by conjugation with dibenzocyclooctyne (DBCO) labeled antibody to generate antibody-conjugated T or NK cells (AbC T cells or AbC NK) (Fig. 1A). To determine the amount of Ac_4_ManNAz needed in the culture media to maximize cell surface azide modification, we cultured activated T cells and NK-92 cells with varying concentrations of Ac_4_ManNAz and detected azides using DBCO-containing fluorophore (DBCO-Fluor 488). Fluor 488-modified cells were quantified using flow cytometry where the fluorescence signal plateaued at 40 μM Ac_4_ManNAz (Fig. 1B). Activated T cell and NK-92 cell viability was measured under these conditions (Figure S1) to determine whether Ac_4_ManNAz was toxic to cells over this range. Both activated T cells and NK-92 cells viability decreased at Ac_4_ManNAz concentrations above 40 μM, and thus, we used 40 μM Ac_4_ManNAz for further T cell and NK-92 azide modifications.

The 14F7hT antibody substrate was prepared by conjugating it to DBCO-PEG_4_-N-hydroxysuccinimidyl ester (DBCO-PEG4-NHS ester) to produce 14F7hT with a labeling ratio of 1–2 DBCO molecules per antibody. DBCO-modified 14F7hT was added to the azide-modified NK-92 and activated T cells for 2 h to produce AbC NK and AbC T cells directed towards Neu5Gc-GM3 positive cells.

Conjugated antibody was detected using anti-human IgG secondary antibody and analysis by flow cytometry (Fig. 1C). We observed a dose-dependent labelling of 14F7hT-DBCO to azide modified activated T cells with a saturating concentration at 4 μM (Figure S2).

To examine the lifetime of 14F7hT on AbC T and AbC NK cells, we cultured activated AbC T and AbC NK cells over 48 h. We detected levels of 14F7hT present on the AbC T and AbC NK cell surface through flow cytometry using an anti-human IgG secondary antibody. Levels of 14F7hT on the cell surface decreased over 48 h with ~31 and 13% of the antibody remaining on the AbC T and AbC NK cells, respectively (Fig. 1D). The loss of antibody on the cell surface was likely due to dilution that occurs during cell division (Figure S3). The number of antibody molecules displayed on AbC T and AbC NK cells was calculated through comparison with beads with known antibody binding capacity. T cells displayed ~380,000 antibody molecules/cell, whereas NK-92 displayed ~110,000 antibodies/cell (Figure S4). Conjugation of 14F7hT to T or NK-92 cell surface did not affect cell viability (Fig. 1E) or proliferative capacity (Figure S5).

### Specific binding of activated 14F7hT AbC T and AbC NK cells to Neu5Gc-GM3 positive cells

We determined whether conjugation of 14F7hT to activated T or NK-92 cells enhanced their interaction with Neu5Gc-GM3 positive cells. The Neu5Gc-GM3 positive L1210 murine lymphocytic leukemia cell line was pre-stained with CellTrace Violet fluorescent dye, and T and NK-92 cells stained with CellTracker Green fluorescent dye. Equal ratios of L1210 target cells and antibody conjugated or unconjugated activated T or NK-92 cells (effector cells) were incubated together for 2 h, and cell clusters were analyzed by flow cytometry. Both antibody-conjugated effector cell types bound to target cells and formed E/T clusters significantly more than unconjugated control activated T and NK-92 cells (Fig. 1F).

### Specific killing of Neu5Gc-GM3 positive target cells by 14F7hT AbC T and AbC NK cells

**Fig. 2:**
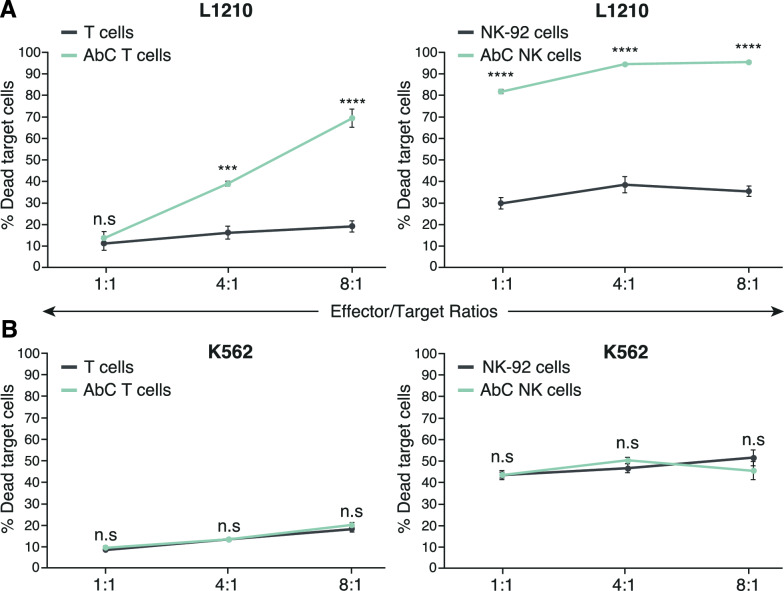
Specific cytotoxicity of activated AbC T and AbC NK cells. T or NK cells or AbC T or AbC NK cells were cultured with antigen-expressing L1210 cells (**A**) or antigen negative K562 cells (**B**). Effector and target cells were cultured for 16 h at different E/T ratios. Cells were stained with 7-AAD and analyzed by flow cytometry with target cells analyzed for their death rates. Data is representative of 3 independent experiments with T cells sourced from 3 different donors. Data represents the mean ± SD (*n* = 3). (Student’s *t*-test, ***p < 0.001 and ****p < 0.0001, *ns* not significant)

We examined the ability of AbC T and AbC NK cells to kill Neu5Gc-GM3 positive target cells relative to untargeted activated T and NK-92 cells. AbC NK and AbC T cells or unconjugated activated T and NK-92 cells were co-cultured with L1210 cells at E/T ratios of 1:1, 4:1, and 8:1 and the viability of L1210 cells was evaluated using flow cytometry. Effector cells were pre-stained with CellTrace Violet fluorescent dye to differentiate them from target cells, and following a 16-h incubation, cell viability was assessed by staining with 7-AAD dye. AbC T cells exhibited significantly enhanced cytotoxicity of Neu5Gc-GM3 positive L1210 cells at 4:1 and 8:1 E/T ratios compared to untargeted activated T cells (Fig. 2A). AbC NK cells also showed enhanced killing effects on target cells over untargeted NK at all E/T ratios tested. In contrast, AbC T and AbC NK cells did not induce significant increases in cell death on off-target control Neu5Gc-GM3 negative K562 cells (Fig. 2B), demonstrating that the AbC T and AbC NK cell killing effect is specific to target cells. Further, we showed that activated AbC T cells conjugated with a non-specific IgG (human anti-EGFR antibody nimotuzumab) showed significantly reduced cytotoxic activity (Figure S6), indicating that the killing effect of AbC immune cells is dependent on the targeting conferred by the 14F7hT antibody.

Even though human cells lack the enzyme to synthesize Neu5Gc, human cell lines cultured with free Neu5Gc can incorporate it into their glycoproteins [32]. To test the activity of AbC T cells against a human cell line, we cultured the human chronic myeloid leukemia K562 cell line with 3 mM Neu5Gc for 3 days and demonstrated 14F7hT-positivity compared to untreated cells (Figure S7A). Similar to L1210 target cells, the Neu5Gc-fed K562 cells showed significantly higher death rates when exposed to AbC T cells compared to control activated T cells at 4:1 and 8:1 E/T ratios (Figure S8). The degree of cytotoxicity, however, was lower for K562 cells fed with Neu5Gc relative to L1210 cells, which could be explained by the lower Neu5Gc-GM3 amount present on the Neu5Gc fed K562 cells (Figure S7B).

To confirm that the observed cytotoxic effect of AbC T and AbC NK cells was due to the activity of effector cells and not the 14F7hT antibody, we conjugated 14F7hT on the surface of K562 cells, which do not have cytotoxic capabilities. We co-cultured 14F7hT AbC K562 cells with L1210 target cells at an E/T ratio of 8:1 for 16 h. Although the antibody was successfully displayed on the surface of K562 cells (Figure S9A),14F7hT AbC K562 cells did not show the ability to lyse target cells (Figure S9B), indicating that the killing effect of activated AbC T cells and AbC NK cells is effector cell-mediated, and not due to the 14F7hT antibody itself.

**Fig. 3:**
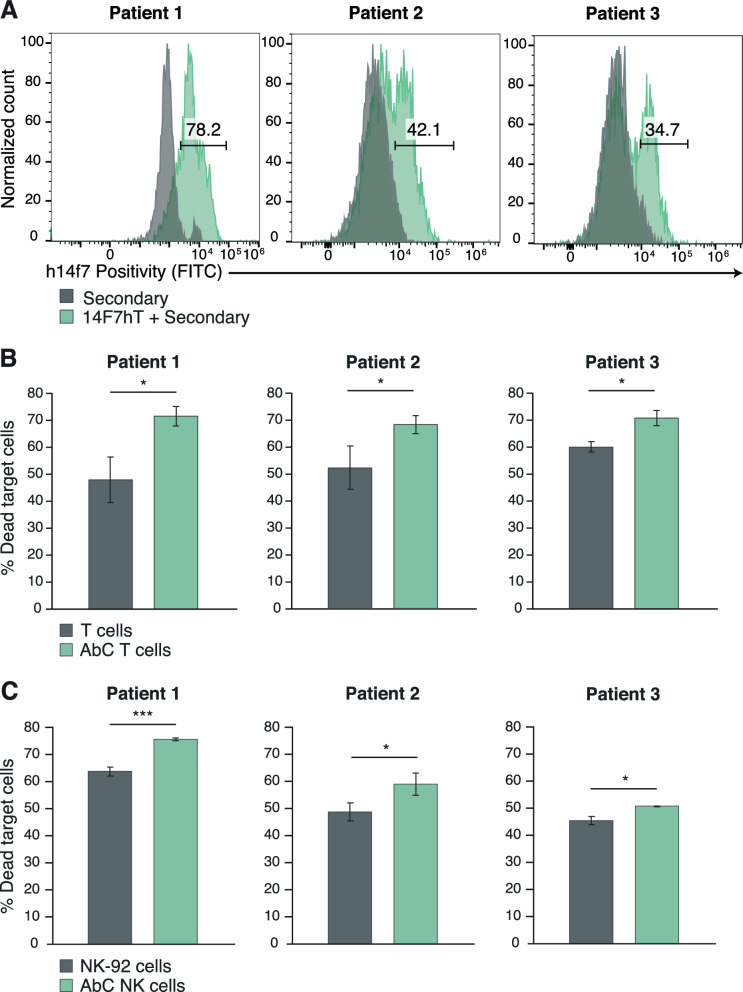
Primary leukemia patient characterization and specific cytotoxicity of activated AbC T and AbC NK cells. **A** 14F7hT positivity of 3 patient primary chronic lymphocytic leukemia biopsies. **B** AbC T or unconjugated T cells and AbC NK or parental NK-92 (**C**) were cultured with primary leukemia cells for 16 h at 8:1 E/T ratio. Cells were stained with 7-AAD and analyzed by flow cytometry with target cells (14F7hT+) analyzed for their death rates. Data represents the mean ± SD (*n* = 3). (Student’s *t*-test, *p < 0.05 and ***p < 0.001, ns, not significant)

To demonstrate the activity of AbC T and AbC NK cells in leukemia biopsies, we examined their activity in chronic lymphocytic leukemia (CLL) patient blood samples. Patient biopsies were assessed for 14F7hT positivity using flow cytometry (Fig. 3A). To assess whether AbC NK and AbC T cells can specifically lyse primary CLL patient cells, we cultured AbC T and AbC NK cells with CLL cells at an 8:1 E/T ratio and cytotoxicity was quantified as previously described. Both AbC T and AbC NK cells displayed increased killing against 14F7hT-positive CLL cells compared to unconjugated cells, showing AbC NK and AbC T cell functionality against Neu5Gc-GM3 positive patient leukemia cells (Fig. 3B-C).

### Activation of 14F7hT AbC T cells by target cells

To explore whether AbC T cells were becoming further activated when exposed to Neu5Gc-GM3 positive cells, we measured the levels of early CD69 and late CD25 activation markers using flow cytometry. Under these conditions, no further changes in activation markers CD69 and CD25 were observed, most likely due to the high expression of these markers after activation with anti-CD3/CD28 antibody beads (data not shown). We next sought to determine if AbC T cells not previously activated with activation beads could become activated in the presence of antigen-positive target cells. First, we determined whether unactivated T cells, which are in a quiescent state, could be conjugated with antibodies, as this would require metabolizing Ac_4_ManNAz and incorporating it into their cellular glycan. T cells isolated from blood were cultured in Ac_4_ManNAz and conjugated with DBCO-14F7hT. Flow cytometry analysis confirmed that unactivated T cells display antibodies on their cellular surface similar to activated T cells (Figure S10).

**Fig. 4:**
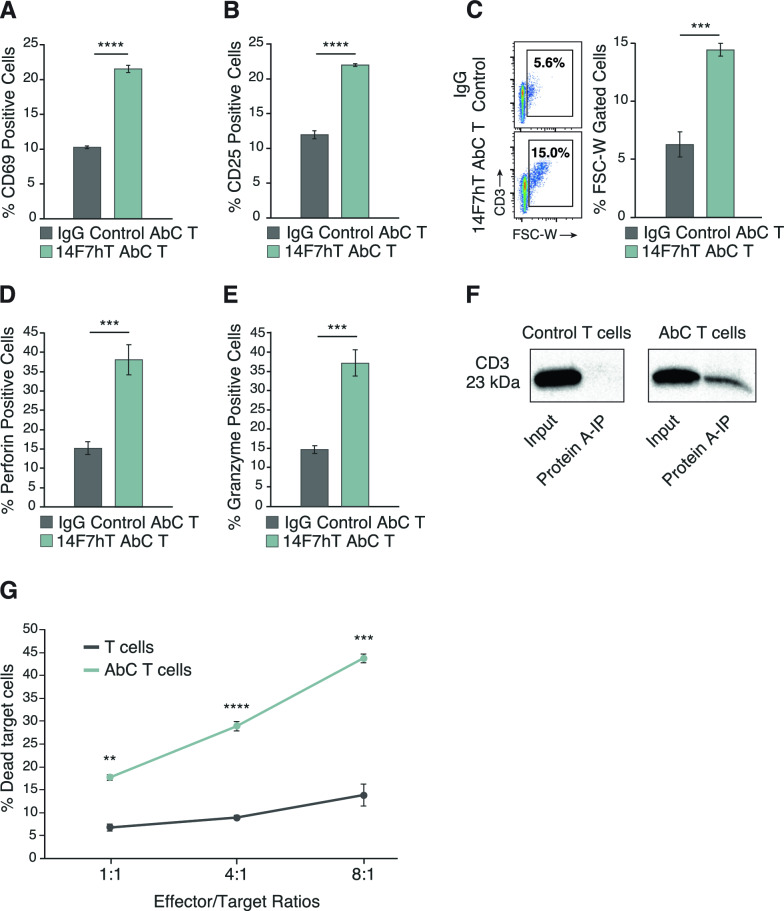
Increase of activation markers and specific killing after interaction of unactivated AbC T cells and target cells. Expression of CD69 (**A**), CD25 (**B**), forward scatter profile (**C**), perforin (**D**) and granzyme (**E**) on IgG control AbC T cells or 14F7hT AbC T cells was measured by flow cytometry after co-culturing with antigen-expressing L1210 cells at 4:1 E/T ratio. **F** Control and AbC T cells were evaluated via co-immunoprecipitation for 14F7hT linkage to CD3. The input and eluted materials were evaluated by Western blot. **G** T or AbC T cells were cultured with antigen-expressing L1210 cells for 16 h at different E/T ratios. Cells were stained with 7-AAD and analyzed by flow cytometry with target cells analyzed for their death rates. Flow cytometry data is representative of 3 independent experiments with T cells sourced from 3 different donors. Data represents the mean ± SD (*n* = 3). (Student’s *t*-test, **p < 0.01, ***p < 0.001 and ****p < 0.0001, *ns* not significant)

To evaluate whether unactivated AbC T cells could become activated in the presence of Neu5Gc-GM3 positive L1210 cells, we exposed AbC T cells and IgG control conjugated T cells to target cells for 16 h and measured levels of CD69 and CD25 using flow cytometry. Unactivated AbC T cells demonstrated a significant increase in CD69 and CD25 activation markers in response to co-culture with target cells compared with control IgG-conjugated T cells (Fig. 4A, B). Activation of T cells increases T cell size in a process known as blastogenesis. This increase in size is quantified by measuring the forward scatter (FSC) of T cells using flow cytometry [33–36]. Unactivated AbC T cells exhibit blastogenesis in response to Neu5Gc-GM3 positive cells, as demonstrated by an increase in FSC compared to IgG control AbC T cells (Fig. 4C). In addition to forward scatter, side scatter (SSC), a measure of cellular complexity/granularity, has been used to measure T cell activation and proliferation [37–39]. Similar to the increase seen in FSC, unactivated AbC T cells show a significant increase in SSC over their IgG-conjugated controls when encountering tumor cells (Figure S11).

T cells employ granule-mediated mechanisms for target cell killing. Therefore, we evaluated the intracellular granzyme and perforin expression of unactivated AbC T cells and control IgG-conjugated T cells when exposed to Neu5Gc-GM3 positive L1210 cells. Unactivated 14F7hT AbC T cells markedly increased expression of both granzyme and perforin when co-cultured with antigen-positive cancer cells compared to IgG control non-activated T cells (Fig. 4D, E).

### Antibodies conjugated to cell surface protein contribute to T cell activation

CD3 is an essential component of the T cell receptor (TCR) and is vital in activating lymphocytes. Conjugation of 14F7hT to CD3 could result in clustering and activation of the TCR. We investigated if 14F7hT on the surface of the T cell is being conjugated to CD3, as the TCR is known to be heavily glycosylated [40, 41]. Activated 14F7hT AbC T cells and unconjugated T cells were subjected to a co-immunoprecipitation assay using Protein A beads, which binds 14F7hT, and co-precipitation of CD3 was detected using Western analysis with an anti-CD3 antibody. CD3 was detected in the co-precipitation with Protein A, confirming the conjugation of 14F7hT to the CD3 domain of the T cell receptor (Fig. 4F).

### Target cell killing by unactivated AbC T cells

Unactivated T cells are not primed for cytotoxic killing; however, we sought to determine if the cytotoxic activity of these unactivated T cells could be activated when conjugated to 14F7hT in the presence of Neu5Gc-GM3 positive cells. Unactivated T and AbC T cells were cultured with L1210 cells as previously described. At all E/T ratios, the unactivated AbC T cells demonstrated enhanced cytolytic activity compared to control unactivated T cells (Fig. 4G); however, the degree of cytotoxicity is less than that of anti-CD3/CD28 antibody bead-activated T cells.

## Discussion

To harness and direct the cytotoxic capabilities of T and NK cells in and MHC and NK-activating receptor-independent manner, antibody-conjugated cells were generated, enabling targeting of tumor cells through cell surface engineering. T and NK-92 cells were engineered to display targeting antibodies through metabolic glycoengineering and SPAAC click chemistry in a simple, time- and labor-efficient manufacturing process. Importantly, generating antibody-conjugated cells does not require genetic engineering, which is necessary for CAR T or NK production, and carries technical limitations such as inefficient gene transfer, inconsistent CAR expression, potential carcinogenicity of gene-edited cells and high production costs [7, 8]. The modification of T and NK-92 cells was highly efficient and all cells were successfully conjugated with antibodies without modifying cellular properties. When armed with antibodies, T and NK-92 cells were able to specifically target tumor cells and form stable E/T clusters.

As cytotoxic proteins such as granzyme and perforin are upregulated upon T cell receptor activation [42–45], we hypothesized that T cells would require pre-activation with anti-CD3/CD28 antibody activation beads for target cell killing. Pre-activated AbC T cells demonstrated significant potency in lysing antigen positive L1210 cells. Using a highly active NK cell line, NK-92, we established that AbC NK cells show target-specific cell killing, even at low E/T ratios of 1:1, whereas AbC T cells only demonstrate significant cytotoxicity at ratios of 4:1. Additionally, AbC NK cells exhibited higher rates of target cell killing compared to AbC T cells, despite displaying significantly less antibody on their cell surface. Both activated AbC T and AbC NK cells were able to significantly lyse antigen-positive primary leukemia cells from patient CLL biopsies, highlighting the potential for clinical translation.

Unactivated AbC T cells underwent T cell blasting and increased expression of activation markers CD69 and CD25 as well as cytotoxic granule-associated proteins granzyme and perforin when exposed to target cells, compared to IgG-control conjugated T cells. Additionally, unactivated AbC T cells were able to elicit target cell killing at all E/T ratios. One hypothesis is that binding of the anchored antibody to antigen could result in T cell receptor clustering, thereby activating the TCR and forming an immunological synapse; therefore, we sought to determine if antibody was being conjugated to a component of the TCR. Using co-immunoprecipitation and Western analysis, we determined that 14F7hT was linked to CD3 and could be activated via antibody binding to antigen.

Although CAR T cell therapy has been a ground-breaking tool in cancer treatment, high rates of toxicities and fatal side effects due to over-proliferation of genetically engineered T cells has limited its adoption in the clinic [3–5]. Recently, non-viral, transient approaches such as mRNA transfection to generate CAR T cells have been developed to improve the safety profile and limit toxicities associated with CAR T therapy [46, 47]. The use of surface-modified T cells would be another method to reduce side effects and would be expected to have favorable toxicity profile compared to gene-edited CAR Ts.

There is a need for developing novel low-cost immunotherapies [48, 49]. The rapid manufacturing of a tumor-targeted AbC T and AbC NK cells without genetic engineering represents a cost-effective approach, whereas the high price and lengthy production time of CAR T and NK has limited its broader use. CAR T cell engineering is most commonly accomplished via lentiviral transduction, which is inefficient in non-activated T cells [50, 51]. Generally, T cell therapies involve prior activation, typically with anti-CD3/CD28 beads, for transduction and expansion purposes [50]. We demonstrated target-specific killing with antibody-engineered T cells without the requirement for pre-activation, which would condense the engineering process from up to 2 weeks for CAR T therapy [52–54] down to 24 h, significantly reducing both the manufacturing time and the cost of production. In addition to cost-savings, reducing the duration between apheresis and engineered immune cell infusion can broaden the application for cellular immunotherapy for patients with rapidly progressing disease, as 20–30% of enrolled patients in several CAR T clinical trials targeted CD19 could not be treated with CAR T therapy due to death from rapid leukemic progression or production failure of the CAR T cells [55, 56].

One challenging limitation of CAR T/NK cell therapy is antigen escape from the development of tumor resistance to CAR constructs targeting a single antigen [57, 58]. Directly modifying the cell surface with antibodies allows for the conjugation of multiple antibodies to the same cell, generating multi-targeted T or NK cells with the possibility of preventing tumor antigen escape. The ease of modifying clinical-grade antibodies and straightforward approach to engineering immune cells provide significant advantages for rapid, personalized cellular immunotherapy.

In conclusion, we demonstrated that activated, non-activated T cells and natural killer cells can be rapidly armed with antibodies, without the need for genetic engineering, endowing them with target specificity to efficiently lyse tumor cells.

## Supplementary Information

Additional file 1.

## Data Availability

No datasets were generated or analysed during the current study.
