# Supplementary material for: Antibody-targeted T cells and natural killer cells for cancer immunotherapy
Source: J Nanobiotechnology. 2024 Oct 18;22:640. doi: 10.1186/s12951-024-02898-3 (PMC11488284; doi:10.1186/s12951-024-02898-3)
Supplement: Supplementary file 1 — Additional file 1. [file 12951_2024_2898_MOESM1_ESM.pdf]

Supporting Information

Antibody-Targeted T Cells and Natural Killer Cells for Cancer Immunotherapy

Ashley R. Sutherland[1], Brijesh Parlekar[2], David W. Livingstone[2], Andrés X. Medina[3], Wendy Bernhard[3], Tays Hernández García[4], John DeCoteau[3], C. Ronald Geyer[3]*

[1]Department of Biochemistry, Microbiology and Immunology, University of Saskatchewan, Saskatoon, SK, S7N 5E5, Canada
[2]Department of Health Sciences, University of Saskatchewan, Saskatoon, SK, S7N 5E5, Canada
[3]Department of Pathology and Laboratory Medicine, University of Saskatchewan, Saskatoon, SK, S7N 5E5, Canada
[4]Center for Molecular Immunology, Havana, 11600, Cuba

*Corresponding author email: clg595@mail.usask.ca

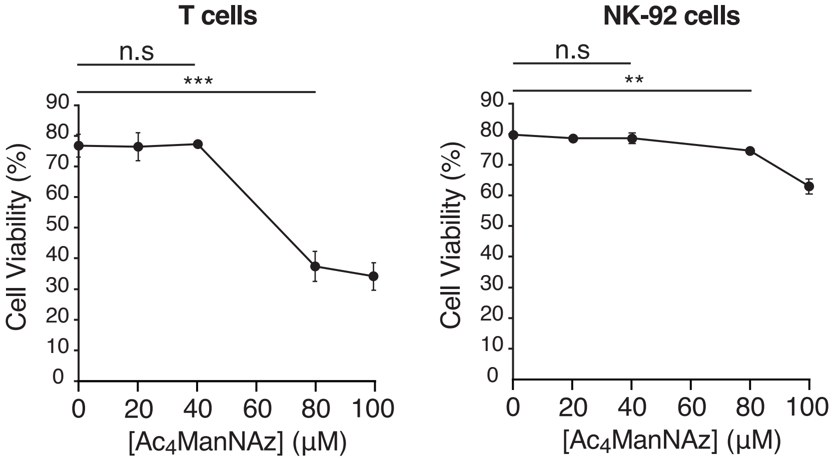

Figure S1. Viability of T and NK cells decrease after 40 µM $Ac_4ManNAz$. Viability of T and NK cells was measured by Trypan blue exclusion after culturing in various concentrations of $Ac_4ManNAz$ for 24 hours.

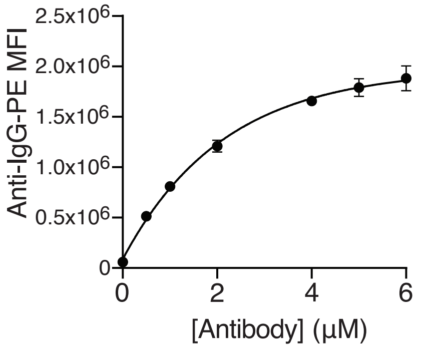

Figure S2. Titration of DBCO-14F7hT on glycoengineered T cells. Glycoengineered activated T cells were conjugated with varying concentrations of DBCO-14F7hT and detected through staining with anti-human IgG-PE via flow cytometry.

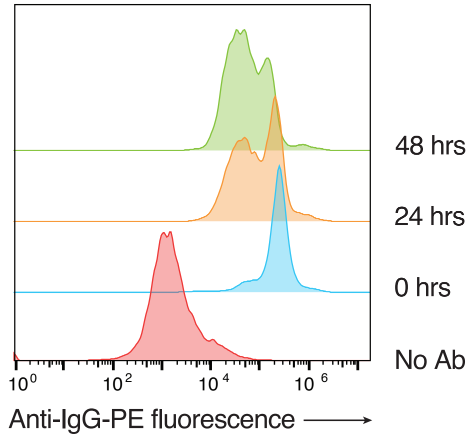

Figure S3.Stability time course study of AbC T cells. 14F7hT was detected on the surface of AbC T cells through staining with anti-human IgG-PE secondary antibody via flow cytometry at time of conjugation, 24- and 48-hour time intervals. Two peaks are present after 24 hours, with the right peak representing the original labeled cells and the left peak showing cells that have most likely lost antibody due to cell division.

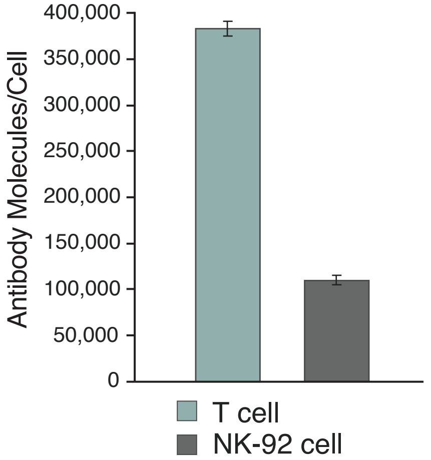

Figure S4. Determination of number of antibodies per T or NK cell. The number of antibody molecules displayed on AbC T or AbC NK cells was calculated through comparison with beads with known antibody binding capacity by flow cytometry after conjugation.

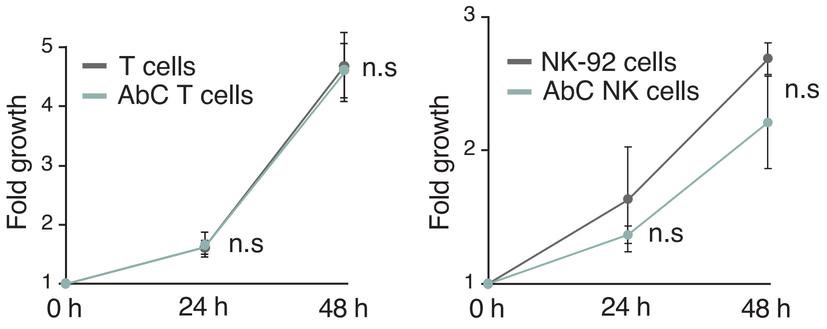

Figure S5. Evaluation of effector cell growth following conjugation with antibody. T cells and AbC T cells and NK cells and AbC NK cells growth was measured over 48 hours by hemocytometer counts.

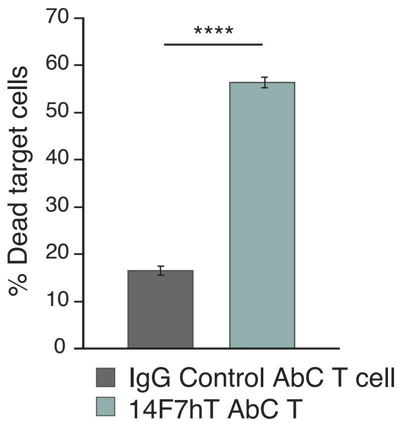

Figure S6. Specific cytotoxicity of activated AbC T cells. Non-specific IgG-conjugated AbC T cells or 14F7hT AbC T cells were cultured with antigen-expressing L1210 cells. Effector and target cells were cultured for 16 hours at 8:1 E/T ratio. Cells were stained with 7-AAD and analyzed by flow cytometry with target cells analyzed for their death rates.

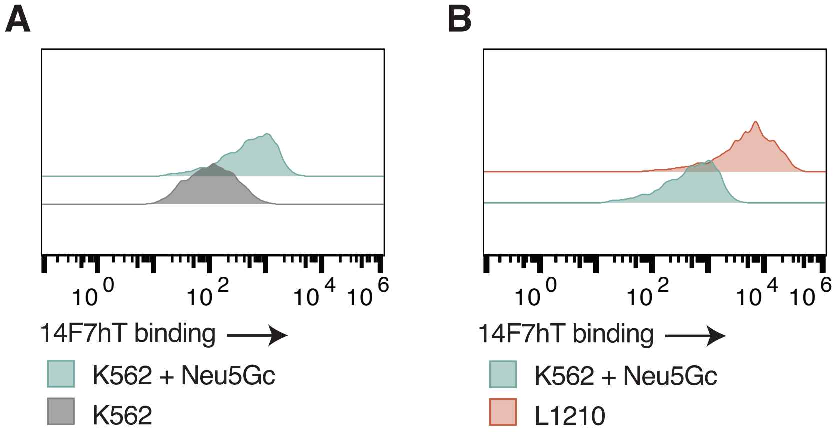

Figure S7. 14F7hT binding to target cells. A) K562 or K562 cultured for 3 days with Neu5Gc (K562+Neu5Gc) or B) K562+Neu5Gc and L1210 were analyzed by flow cytometry to determine level of 14F7hT binding.

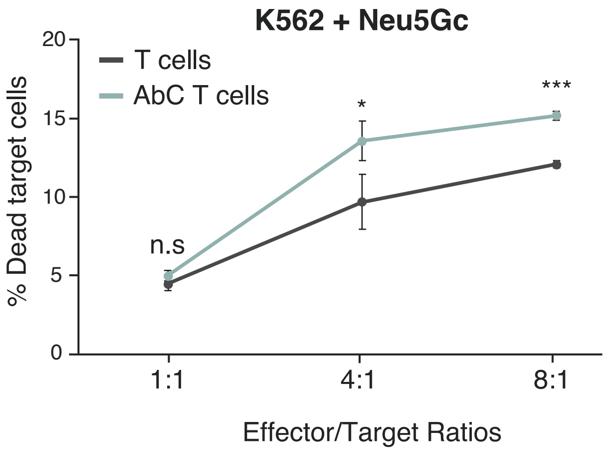

Figure S8. Specific cytotoxicity of activated AbC T cells against K562 cultured with Neu5Gc antigen. Activated T or AbC T cells were cultured with K562 cells fed with Neu5Gc antigen. Effector and target cells were cultured for 16 hours at different E/T ratios. Cells were stained with 7-AAD and analyzed by flow cytometry with target cells analyzed for their death rates.

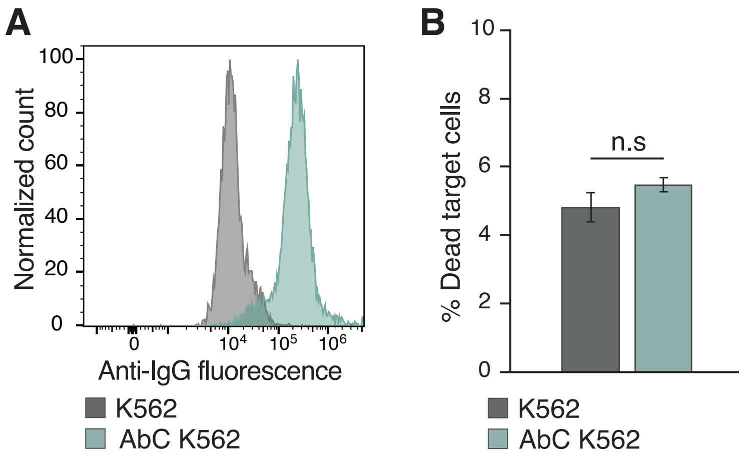

Figure S9. 14F7hT conjugated-K562 lymphoblast cell line does not exhibit enhanced killing of target cells. A) Conjugation of 14F7hT-DBCO to glycoengineered K562 cell line as detected by anti-human IgG-PE, analyzed by flow cytometry. B) K562 cells or 14F7hT conjugated K562 cells were cultured with antigen expressing L1210 cells for 16 hours at 8:1 effector/target cell ratio. Cells were stained with 7-AAD and analyzed by flow cytometry with target cells further analyzed for their death rates.

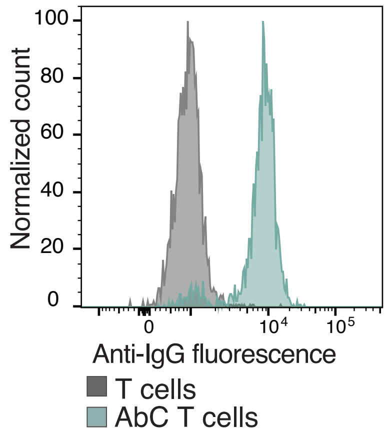

Figure S10. Antibody conjugation to non-preactivated T cells. Flow cytometry histograms showing conjugation of DBCO-14F7hT to surface of glycoengineered non-preactivated T or AbC T cells through staining with anti-human IgG-PE.

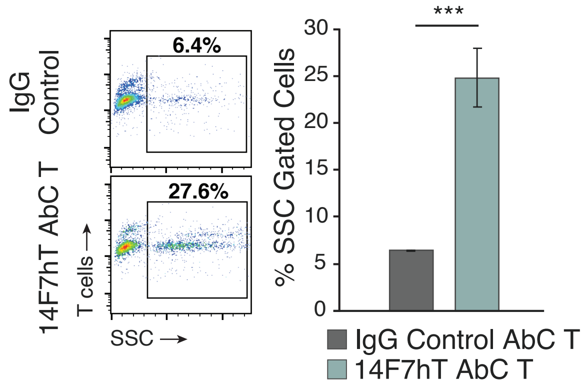

Figure S11. Non-preactivated AbC T cells demonstrate an increase in side scatter compared to IgG-conjugated controls when encountering antigen. IgG-control AbC T cells or 14F7hT AbC T cells were cultured with antigen-positive L1210 cells for 16 hours. Side scatter of T cells was measured by flow cytometry.
